# SERS Sensors with Bio-Derived Substrates Under the Way to Agricultural Monitoring of Pesticide Residues

**DOI:** 10.3390/bios14120573

**Published:** 2024-11-26

**Authors:** Kseniya V. Serebrennikova, Nadezhda S. Komova, Anatoly V. Zherdev, Boris B. Dzantiev

**Affiliations:** A.N. Bach Institute of Biochemistry, Research Center of Biotechnology of the Russian Academy of Sciences, Leninsky Prospect 33, 119071 Moscow, Russia; ksenijasereb@mail.ru (K.V.S.); nad4883@yandex.ru (N.S.K.); zherdev@inbi.ras.ru (A.V.Z.)

**Keywords:** SERS substrates, Raman spectroscopy, natural biomaterials, biopolymers, aptamers, pesticides, photosystem-inhibiting herbicides

## Abstract

Uncontrolled use of pesticides in agriculture leads to negative consequences for the environment, as well as for human and animal health. Therefore, timely detection of pesticides will allow application of measures to eliminate the excess of maximum residue limits and reduce possible negative consequences in advance. Common methods of pesticide analysis suffer from high costs, and are time consuming, and labor intensive. Currently, more attention is being paid to the development of surface-enhanced Raman scattering (SERS) sensors as a non-destructive and highly sensitive tool for detecting various chemicals in agricultural applications. This review focuses on the current developments of biocompatible SERS substrates based on natural materials with unique micro/nanostructures, flexible SERS substrates based on biopolymers, as well as functionalized SERS substrates, which are close to the current needs and requirements of agricultural product quality control and environmental safety assessment. The impact of herbicides on the process of photosynthesis is considered and the prospects for the application of Raman spectroscopy and SERS for the detection of herbicides are discussed.

## 1. Introduction

Pesticides are substances or mixtures of chemical or biological origin intended to control pests, weeds, and external parasites of plants in order to maintain or increase crop yields. The total amount of pesticides used worldwide exceeds three billion kilograms per year, of which only a small percentage (no more than 1%) is used for effective pest control, while the remaining large part penetrates into uninfected plants or remains in the environment [1,2]. Through extensive production and increased use of agricultural pesticides, food crop yields worldwide have almost doubled [3]. Despite the benefits for agricultural development provided by the use of pesticides, their use is associated with certain problems. Pesticide residues in food products pose a potential danger to all living organisms, their ingestion leads to the development of acute or chronic poisoning depending on the dose of the toxin. To control pesticide residues in food products, maximum residue levels (MRLs) have been established by regulatory agencies [4]. Pesticides are divided into several main groups, namely herbicides used to destroy weeds; insecticides and fungicides aimed at combating insect pests and pathogenic fungi, respectively; zoocides used against harmful warm-blooded animals; bactericides aimed at destroying bacteria; virucides used to destroy harmful viruses [5]. The largest segment of the pesticide industry is herbicides, which account for almost 48% of the total use of pesticides [6]. Although global regulation of MRLs in food remains a challenge, on average, extremely low concentrations of pesticides are allowed in food [7]. Therefore, the choice of reliable and rapid methods for determining pesticide residues is an important task for consumer’s protection, as well as for the assessment of environmental pollution.

Main conventional methods for determining pesticide residues in environmental objects and agricultural products include gas chromatography–mass spectrometry and high-performance liquid chromatography [8,9]. Compared to these laborious, expensive and time-consuming methods, sensors with different detection techniques represent promising tools for simple on-site monitoring. Thus, the latest progress is described in reviews covering immunosensors [10,11], electrochemical and optical sensors [12,13,14] for pesticide detection. While advances in optical and electrochemical sensors have improved their performance, other detection methods have emerged that promise additional benefits such as reduced analysis time, simplified testing procedures, on-site sampling, portability, and low costs.

Raman scattering is a phenomenon of inelastic light scattering by molecular systems with energy unique to the given structure and scattering intensity directly proportional to the number of molecules [15]. Raman spectroscopy is a non-destructive qualitative and quantitative method of analysis [16]. However, despite the advantages of Raman spectroscopy, including wide availability on glass, solvent, water, etc., as well as minimal sample preparation without much sample damage, low Raman signals limit the applicability of this technique in food control. In turn, recent developments in SERS substrates and design of sensors expand the potential of Raman-based techniques for wide application in different areas where timely identification and sensitive detection of toxins is in demand.

**Figure 1:**
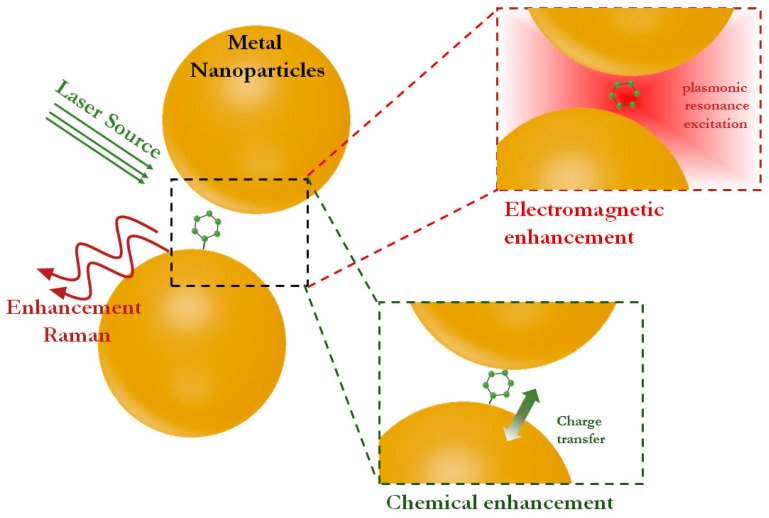
Illustration of the mechanisms that provide SERS enhancement.

Surface-enhanced Raman scattering is based on the electromagnetic and chemical interaction between the excitation laser, the target analyte and the plasmonic substrate, resulting in a significant enhancement of the Raman signals of the analyte (Figure 1). Raman enhancement can reach 10^14^ when the molecules of interest are located in so-called “hot spots”, where a combination of electromagnetic enhancement, charge transfer and plasmonic resonance excitation mechanisms are observed [17]. However, the dominant contribution to the enhancement of Raman scattering is made by electromagnetic fields (up to 10^9^) provided by the excitation of electron oscillations on the surface of the plasmonic substrate (i.e., localized surface plasmon resonance). A smaller contribution (from 10^5^ to 10^7^) is provided by chemical enhancement, realized due to the interaction of the target molecule with the SERS substrate accompanied by charge transfer and a change in the polarizability of the molecule [15,18]. Therefore, the design of suitable SERS substrates that combines and maximizes all possible enhancement mechanisms allows achieving the lowest detection limits while maintaining the advantages of Raman spectroscopy.

**Figure 2:**
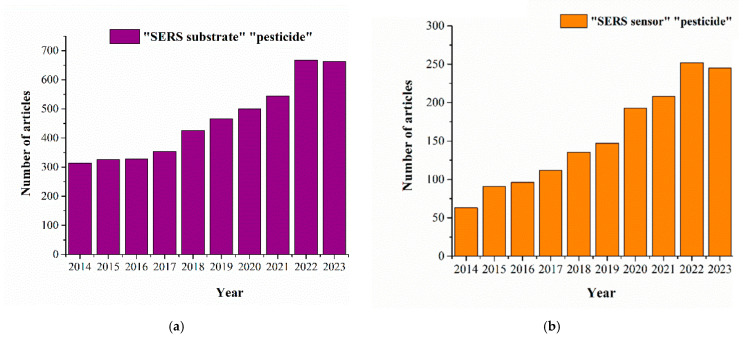
The number of publications is displayed as a function of year using the search query “SERS substrate pesticide” (**a**) or “SERS sensor pesticide” (**b**) in PubMed for the last 10 years.

Currently SERS-based techniques for pesticide detection can be divided into two directions (see Figure 2)—the development of SERS substrates with demonstration of the capabilities for testing various agricultural products, and the development of SERS sensors with the ability to selectively detect the target analyte in complex samples. The first direction focuses on combining the latest advances in nanotechnology and materials to fabricate effective SERS substrates for highly sensitive and reproducible assays [19,20]. The second direction focuses on increasing the selectivity of pesticide detection by additionally using receptor molecules (antibodies, aptamers, molecular-imprinting polymers) and expanding the assay formats [21,22].

In recent years, tremendous progress in the design of SERS substrates applied in food control [23,24], biochemical sensing [25,26], etc. [27,28], has been observed. The most common enhancing metals for constructing SERS substrates are silver and gold due to their unique plasmonic properties, which are tuned by varying the size and morphology of the nanoparticles [29,30,31]. SERS detection is based on either mixing a colloid of noble metal nanoparticles with the target analyte or depositing the analyte on a plasmonic substrate [32]. To evaluate the efficiency of SERS substrates, the enhancement factor (EF) including electromagnetic and chemical/charge transfer mechanisms is estimated [18]. On average, the EF achieved on the surface of noble metals does not exceed the range of 10^6^~10^11^. Despite the successful development of SERS substrates summarized in several reviews [23,24,25,26,27,28], the design of efficient SERS substrates with uniform “hot spot” distributions that combine advanced nanomaterials and engineering technologies to address specific challenges remains an important research area. In particular, SERS applications in agriculture include the detection of trace amounts of pesticides in various matrices, where efficient and rapid extraction of target analytes is advanced option.

Recently, the attention of researchers has been attracted by the fabrication of flexible SERS substrates allowing analysis from nonplanar or curved surfaces [20,33]. Particularly significant among these strategies is the use of bioinspired materials that have a natural rough and hydrophobic surface with special micro- and nanostructures (leaves, petals, wings etc.), as well as biopolymers (chitosan, cellulose). The review highlights the application of biomaterials modified with SERS-active plasmonic structures for the detection of pesticides. In addition, the prospects for designing sensor systems based on biomaterials or supramolecular cell complexes as receptors for the identification of photosynthetic-inhibiting herbicides are considered.

## 2. Exploring the Diversity of Biomaterials and Design of Biocompatible SERS Substrates

The integration of nanomaterials with unique micro/nanostructure of natural or bio-derived materials has shown great potential for obtaining cheap, flexible and biocompatible SERS substrates. Leaves, flower petals, insect wings or mussel shells have naturally occurring structures with nanoarrays or nanopillars, which, when coated with a silver or gold layer, provide the formation of “hot spots” and enhancement of the SERS signal. Moreover, the hydrophobic surface of biological materials ensures that the droplet containing the analyte is concentrated in a narrow region after drying, which also improves the SERS-based assaying for low concentrations of target compounds [34]. Finally, regular microstructures of biomaterials promote a uniform distribution of plasmonic nanostructures, which ultimately increases the reproducibility of the SERS signal. Recent developments of SERS substrates based on various biological materials and analytical performance (limit of detection and reproducibility of SERS signal) are summarized in Table 1. Important criteria for assessing the effectiveness of SERS substrates are the enhancement factor and reproducibility of SERS measurements. Reproducibility, which reflects the variability of the signal at different spots of the same substrate, is usually estimated as the relative standard deviation (RSD) of the SERS signal and depends mainly on the distribution of hot spots on the substrate. Relative standard deviation values of less than 20% are considered acceptable for SERS substrates and indicate good reproducibility of the SERS signal [35,36].

There are several techniques for producing metal layers on hydrophobic surfaces of biomaterials. Self-assembly of metal nanostructures is the most common and simple technique for obtaining SERS substrates [39,40,41,44,68]. For example, self-assembly of Au@Ag NPs on a mussel shell yielded a substrate with an estimated EF of 1.02 × 10^7^ for Rhodamine 6G (Rh6G) [40]. Dong et al. developed an efficient SERS bio-substrate by first sputtering 5 nm Au nanoparticles and then depositing Rh6G-loaded Au@Ag nanocubes on cicada wings fixed on the silicon wafer via a three-phase self-assembly process [41]. The obtained three-phase self-assembled substrate demonstrated high sensitivity with a detection limit of 5 × 10^−9^ M for Rh6G. Another effective deposition technique that provides a regular arrangement involves sputtering of metal nanoparticles on the biomaterial surface [37,38,43]. Thus, Sharma et al. fabricated a SERS substrate on *Canna generalis* leaf support by sputtering a thin mirror-like Au film followed by self-assembly of AuNP aggregates [37]. The combination of sputtering and self-assembly techniques provided a high enhancement capacity of the substrate due to plasmonic interactions of the nanoparticle aggregates with Au in the thin film. The calculated EF for the Rh6G droplet deposited on the SERS substrate was 3.5 × 10^5^. Arnob et al. demonstrated the potential of using a readily available consumable material, eggshells, as a natural support [43]. In this work, three areas of eggshell (outer shell, inner shell, and shell membrane) were coated with different thicknesses of Au by magnetic sputtering. For membranes with optimal Au layer thicknesses, the enhancement factors for benzenethiol were calculated to be 6 × 10^6^, 1.8 × 10^6^, and 1.5 × 10^5^ for outer shell (80 nm Au), inner shell (80 nm Au), and shell membrane (100 nm Au), respectively. An alternative approach was implemented to prepare a hybrid bio-substrate based on AgNPs-modified *Mytilus coruscus* shell by physical vapor deposition followed by graphene oxide dip coating [42]. The physical vapor deposition method allowed achieving a dense organization of nanoparticles with gaps of approximately 3 nm on the substrate surface. In addition, coating the substrate with a graphene oxide layer resulted in a significant enhancement of the SERS due to the involvement of a chemical enhancement mechanism, maintaining the stability of the metal layer, and concentrating the analyte in the gaps (in the “hot spots” of the substrate). The effectiveness of the substrate proposed in this work was confirmed by a high EFr for Rh6G equal to 2.4 × 10^7^.

As a low-cost and flexible SERS substrate that can adapt to the uneven surface of the target sample in terms of wrapping and wiping the attention of researchers has been drawn to biopolymers. They are promising matrices for incorporating nanoparticles into the structure or depositing nanoparticles on the surface of the material. In addition, flexible biopolymers enable the design of drop-and-dry, collection/swabbing, and wearable/attachable SERS sensors [69]. Among biopolymers, summarized in Table 1, chitosan [47,48,49,50,51] and cellulose-based materials [45,52,56,60,70,71] have proven to be the most interesting for SERS applications. Chitosan, obtained by deacetylation of chitin, acquires functional hydroxyl groups, which, together with its own amino groups, ensure the inclusion and stability of metal nanoparticles in the matrix [72]. In addition, chitosan exhibits strong affinity for metals, molecules, macromolecules, etc., which eliminates the need to use other receptor molecules [73,74]. For example, Puente et al. used chitosan to capture analyte molecules by coating nanoparticles deposited on aluminum foil [48]. In this work, various plasmonic nanostructures were considered, including Ag nanospheres, Ag nanocubes, Au nanospheres, and Au nanorods, and the conditions for the formation of a chitosan film on the surface of the nanoparticles were varied. Compared with the nanostructures considered in the study, the use of Ag nanocubes coated with a chitosan film allowed achieving limit of 4-aminothiophenol detection equal to 1 nM. Another approach for obtaining biomimetic chitosan-based SERS substrate is described in [50] where gold nanoparticles were embedded into a chitosan matrix by in-situ synthesis in a biopolymer medium. Here, chitosan chains promoted the reduction of Au^3+^ ions and served as centers for the formation of Au nuclei. The enhancement factor estimated for 4-mercaptobenzoic acid using Au-decorated chitosan film was 1.4 × 10^7^. Vafakish et al. functionalized chitosan with S-acetylmercaptosuccinic anhydride to form free thiol groups [49]. The functionalized chitosan then served as a matrix for the immobilization of AgNPs, resulting in a homogeneous distribution of non-aggregated AgNPs in the composite. Evaluation of the performance of the SERS substrate showed EF for methylene blue (MB) calculated to be 1.3 × 10^8^, and the possibility of substrate reusing the after 3 washing cycles. Wang et al. proposed a porous 3-D chitosan foam structure embedded with Ag nanoparticles as a flexible SERS substrate that enables swabbing of the analyte from the surface or adsorption of target molecules from solution [51]. The chitosan solution, upon freeze-drying, formed a foam with an open-cell structure that bound the nanoparticles via free amino groups. The resulting SERS substrate allowed Nile Blue A detection down to 36 pg when the sample was drop casted, whereas the swabbing test revealed a detection limit of 5 pg. Performance testing of AgNP-embedded chitosan foam by adsorption of the analyte from solution showed a detection limit of 10 ppb for Rh6G.

A biopolymer that is somewhat less common in the described developments compared to chitosan but has the same advantages for obtaining flexible porous SERS substrates with high sorption capacity is alginate [62,63,64]. For example, Fu et al. prepared Au nanoparticles embedded sponge from sodium alginate gel through freeze-drying technique [61]. Analysis of the Raman spectra of Rh6G probe deposited on the SERS sponge revealed a detection limit of 0.1 nM. By combining cationic chitosan and anionic sodium alginate, Guo et al. synthesized a polyelectrolyte complex without using additional cross-linkers [75]. Incorporation of Ag nanocubes into chitosan-alginate gel followed by freeze-drying resulted in the formation of a flexible SERS substrate with a porous network structure. The enhancement factor evaluation by application thiram on the surface of the SERS substrate showed a value of 1.34 × 10^6^.

In addition, the attention of researchers is attracted by an alternative design of SERS substrates for direct detection of small molecules in complex samples, which is a microgel containing Au or Ag nanoparticles [65,76]. Microgels enable detection of low molecular weight analytes due to the presence of a large number of water-swellable networks with uniform cell sizes, which prevents the penetration of interfering macromolecules. Thus, Lin’s group [65] synthesized Au nanobipyramid@Ag@hyaluronic acid microgel through two-step process including preparation of Au nanobipyramid @Ag–NH_2_ nanoparticles and reaction of activated hyaluronic acid and amino-modified nanoparticles. The SERS effectivity of the Au nanobipyramid@Ag@hyaluronic acid microgel was evaluated using Rh6G and demonstrated EF of 1.2 × 10^8^.

Another common biomaterial that offers advantages of flexibility and biocompatibility is cellulose [77]. Cellulose is a fibrous, renewable biomaterial consisting of D-glucose monosaccharide residues linked by β-1,4 glycosidic bonds. The reactivity of cellulose is determined by such factors as steric effects/hindrances of functional groups and its supramolecular structure. Cellulose has many functional hydroxyl groups, which allows this material to be used as a reducing or stabilizing agent, as well as a carrier of plasmonic nanostructures in the manufacture of SERS substrates. Along with the listed advantages, the availability of the biopolymer, the possibility of modifying the surface and introducing functional groups, and the absorbency of this material are also noted. The advantages of cellulose materials, being taking together, make it more attractive in SERS detection of pesticide residues than other materials [78]. Cellulose at the nanostructure level is finding wider application caused by its exceptionally high mechanical properties with tunable surface chemistry and specific optical properties [79]. There are three main types of nanocellulose, namely nanocrystalline cellulose, nanofibrillated cellulose and bacterial nanocellulose, which differ from each other in morphology, size and crystallinity. Cellulose nanofibrils have good mechanical properties, high strength, large specific surface area, and typical dimensions of 3–15 nm in diameter and 1–3 μm in length [80]. The combination of cellulose nanofibrils with metal nanostructures demonstrated the effectiveness of the resulting hybrid material as a SERS substrate with high functionality (flexibility and mechanical stability) and SERS performance [53,54,70]. Thus, Sun et al. developed SERS wipes based on cellulose nanofiber, Ag nanoparticles, and Au nanostars [55]. It was shown that the use of cellulose nanofibers, which have a positive charge, leads to uniform adsorption of the nanoparticles, resulting in an increase in the SERS signal compared to Ag nanoparticles/Au nanostars-modified SERS substrate. The analytical enhancement factor calculated for 4-ATP using cellulose nanofiber-based substrate was about 10^4^. The unique properties of bacterial nanocellulose, such as high porosity, water-holding capacity, strength and biocompatibility make it an attractive and promising material for creating SERS substrates [81]. For example, Li et al. prepared substrate based on bacterial nanocellulose enriched with Ag nanospheres by in-situ synthesis of nanoparticles (“silver mirror” reaction), followed by drying the hydrogel substrate of bacterial nanocellulose-Ag nanosphere (“volume shrinkage” treatment) [71]. The resulting flexible substrate was characterized by stability and high “hot spot” density with enhancing activity estimated at 1.03 × 10^9^ for 4-mercaptobenzonitrile.

Growing interest in the use of readily available materials has led to the emergence of various methods for processing cellulose to produce hydrogels and aerogels. The three-dimensional and porous structure of hydrogels/aerogels provides the inclusion of plasmonic nanoparticles, and the dense packing of nanoparticles in turn leads to excellent SERS properties of the substrates [82]. Thus, Ge et al. [45] synthesized flower-like ZnO particles by mineralization of cellulose followed by photocatalytic growth of Ag nanoparticles in a ZnO-modified cellulose aerogel matrix. The resulting flexible cellulose aerogel nanocomposite with embedded ZnO@AgNPs demonstrated an enhancement factor of 4.8 × 10^7^ calculated for Rh6G. To organize metal nanoparticles in large arrays, a combination of hierarchical MoS_2_-microspheres and cellulose acetate hydrogel as supports of cauli-flower-like Au nanoparticles is proposed by Qiu’s group [58]. Here, Au nanoparticles grown in the media containing hierarchical microspheres provided a huge enhancement effect due to the many formed nanogaps, allowing detection of up to 5 × 10^−14^ M Rh6G and MB in aqueous solution. The result of assembling of MoS_2_-microspheres modified with cauliflower-like Au nanoparticles on the hydrogel using a vacuum filtration system was the design of a flexible SERS substrate capable of detecting MB up to 10^−12^ M. Further, transparent nanopaper SERS substrate obtained by drying bacterial cellulose hydrogel with incorporated Au nanoparticles was proposed by Zhou et al. [57]. Testing the performance of nanopaper-based SERS substrate demonstrated the ability to detect low analyte concentrations, estimated down to 10^−10^ M for Rh6G. The porous structure of bacterial cellulose aerogel with dense packing of nanoparticles proved to be an effective SERS substrate for the detection of 2, 4, 6-trinitrotoluene [83]. In this study, Au nanorods coated with Ag nanocubes were modified with p-aminobenzenethiol and combined with polyeth-yleneimine-modified aerogel to improve the SERS selectivity and substrate loading capacity, respectively. The detection limit of 2,4,6-trinitrotoluene achieved using this SERS substrate was 8 × 10^−12^ g/L, and the enhancement factor was up to 1.87 × 10^8^. In summary, RSDs of the SERS signal for most of the considered bio-derived substrates varies from 2.82% to 17.7%, indicating the possibility of using these substrates in analytical applications.

Scaling up SERS substrates based on bio-derived materials is limited by mechanical stability of natural biomaterials as well as batch-to-batch variability of SERS substrates. Since inexpensive, efficient and easy to fabricate SERS substrates are important for practical applications, improving the mechanical stability, durability and efficiency of such natural biomaterials is a future research objective. SERS substrates based on biopolymers, in turn, demonstrate better stability, flexibility, ease of sampling and low costs. Compared with conventional rigid or colloidal SERS substrates, where tedious and destructive sample pretreatments are required for analysis, flexible SERS substrates are adaptable to uneven surfaces and have high absorbability due to the porous structure. When combined with portable or handheld Raman spectrometers, flexible biopolymer-based substrates represent a very attractive technique for detection of pesticides in various samples.

## 3. SERS Detection of Pesticides Using Biomaterial-Based Substrates

The unique morphology of biomaterials with a special natural surface relief attracts special attention due to a number of advantages for the practical application of the SERS method, including a large surface area, uniform distribution of plasmonic nanostructures, high sorption properties of natural materials and flexibility. The use of these SERS bio-substrates is especially relevant for ensuring food and environmental safety, where accurate detection of low concentrations of hazardous pollutants such as pesticides is required. However, the detection of pesticides in food and agricultural samples differs significantly from the analysis of reference materials due to the complex composition of the samples, which can affect the SERS spectra. Current developments of SERS substrates and trends in SERS-based techniques make it possible to cover the tasks of detecting pesticides in a wide range of samples. It is worth noting that the use of universal SERS substrate for the analysis of different pesticides is a favorable choice for pesticide management in agriculture; however, the analytical performance of SERS substrate will vary from analyte to analyte. As was mentioned in the Section 1, the main contribution to the enhancement of the Raman signal is made by the electromagnetic enhancement, which is indeed independent of the nature of the analyzed molecules. However, in addition to the electromagnetic enhancement, it is necessary to consider the structural characteristics of the target analytes, since they do not necessarily have the same orientation on the nanoparticle surface, as well as the interaction of the pesticide molecule with the substrate and the accessibility of their complex during SERS measurements [84]. Therefore, when analyzing a particular pesticide, it is important to consider the parameters of the SERS substrates, including plasmonic properties, design, nature and morphology, on one hand, and the structural features of the target analyte, such as chemical bonds and vibrational properties of functional groups, on the other hand.

SERS techniques for pesticide detection can be divided into two main strategies: (i) in situ detection based on the deposition of metal nanoparticles directly on the surface of the sample followed by SERS spectra registration; (ii) immobilization of plasmonic nanostructures on the surface of a flexible or solid substrate material to combine sampling and analysis of the target analyte.

Most developments of SERS bio-substrates are used for rapid analysis of pesticides in fruits, vegetables or beverages (Table 2). There are several approaches to SERS-based pesticide analysis, including direct analysis of pesticides on the surface/peel without preliminary sample preparation [81,85,86,87,88,89], as well as analysis of liquid samples [90].

Application of flexible SERS substrates for the detection of pesticides and their residues on the surface of fruits or vegetables are relatively widespread [81,85,86]. Sample preparation for pesticide analysis using flexible SERS substrates usually involves pre-spraying the sample surface with ethanol or water, pressing the substrate for several minutes to perform extraction, and finally recording the SERS spectra. Shi et al. developed a flexible and stable biomimetic SERS substrate obtained by magnetron sputtering of gold nanoislands onto dragonfly wings, with the calculated enhancement factor for Rh6G being 2.8 × 10^6^ [85]. The developed SERS bio-substrate was used for the analysis with direct detection of cypermethrin traces on tomato peel with a detection limit of up to 10^−3^ ng/cm^2^. Currently, researchers have been attracted by the use of cellulose-based biopolymers for the production of flexible SERS substrates due to their optical transparency and nanofibrous structure [77,78]. Parnsubsakul et al. detected methomyl on the surface of orange and apple with a sensitivity of up to 3.6 × 10^−7^ M using a fully biodegradable SERS substrate based on silver nanoparticles and bacterial nanocellulose [81]. In turn, Zhang et al. managed to achieve nanomolar concentrations for detecting thiram on grape skin using a SERS sensor also based on bacterial nanocellulose decorated with silver nanorods [87]. The fabrication of flexible SERS substrates based on hydrogels of various compositions is also relevant. Sodium alginate [64], chitosan [75], and gelatin [67,91] are successfully used to obtain gels with porous three-dimensional mesh structures. The prospects for using such materials as SERS substrates are due to their large surface area, which allows adsorbing greater amounts of target molecules on them. Thus, Guo et al. developed a flexible SERS substrate, which is a sodium alginate–chitosan composite gel embedded with silver nanocubes, for the detection of the pesticide thiram in apples [75]. The flexible porous structure of the composite gel contributed to effective contact with target molecules, which made it possible to detect thiram in apples with a detection limit of up to 0.055 mg/L without labor-intensive pre-treatment. Similarly, Qi et al. used sodium alginate to prepare a porous flexible hydrogel SERS substrate with embedded gold and silver core-shell nanoparticles (Au@Ag NPs). The use of Au@Ag NPs allowed for a significant enhancement of the Raman signal due to the formation of nanogaps inside the nanoparticles, thereby increasing the sensitivity of thiram detection to 10^−10^ M [64]. Wang et al. developed a simple approach to obtain a SERS substrate based on a gelatin gel sheet with adjustable nanogaps between silver nanoparticles, and as a result, a uniformly distributed large number of hot spots. The synthesized SERS substrates were used to detect the fungicide malachite green in lake water with a detection limit of 10^−9^ M [67].

The use of SERS bio-substrates for the analysis of liquid samples such as juices is also a promising alternative analytical method due to their homogeneous matrices and, consequently, more uniform distribution of target pesticides compared to analysis on sample peels. However, the analysis of liquid samples in some cases requires preliminary extraction of the target substance and/or its concentration. As a rapid and simple sample pretreatment technique, the QuEChERS (quick, easy, cheap, effective, rugged and safe) methods [92] are widely used to remove interfering components and improve the determination efficiency. Thus, the QuEChERS method allowed the assaying trace amounts of the thiram in peach, apple, and grape juices with detection limits of 0.036 ppm, 0.044 ppm, and 0.044 ppm, respectively [13]. The SERS method has high potential for rapid and sensitive detection of pesticides in various food products, but the complexity of food matrices requires separation of the target analyte by extraction and/or purification. The wide range of approaches described currently makes it difficult to choose a universal method.

Unfortunately, the detection of pesticides in food products does not cover the tasks of controlling the spread of contamination. Since the plant surface is the primary reaction medium for pesticides, it is extremely important to control their content during the growth of agricultural crops in order to prevent the abuse of toxic substances. Application of in situ SERS analysis to assess the amount and extent of pesticide accumulation directly from the plant surface is a promising solution for this task. A general approach of in situ SERS detection is to deposit plasmonic nanostructures directly on the plant surfaces. Thus, lotus leaf mastoid [93], capsicum, celery and cole [94], tea leaves [95], soybean leaves [96], spinach leaf [97] have been successfully applied as SERS bio-substrates for deposition of plasmonic nanostructures, such as Ag micro/nanoarrays [93], Ag nanoislands [94], Au nanoparticles of different sizes [95,96,97]. The developed substrates also represent a bioreceptor system, which allowed not only to evaluate the accumulation of pesticides in plants, but also to study interactions on the surface and in plant tissues by non-destructive method. Thus, Liu et al. proposed a universal and simple method for in situ detection of pesticides by controlled growth of silver nanoislands on the surface of artificial and natural materials using a modified Tollens method [94]. This approach was successfully tested for the detection of paraquat and fenthion on the surface of pepper, celery and cabbage with concentrations up to 10^−9^ M and 10^−8^ M, respectively. In addition, application of in situ SERS provided an opportunity to assess the rate and extent of pesticide penetration into plant leaves. Hou et al. studied the ability of the pesticide ferbam to penetrate into tea leaves by applying gold nanoparticles of different sizes and recording SERS spectra [95]. It was found that in 1 h, gold nanoparticles penetrated 1/3–1/2 of the leaf thickness (190 μm). In addition, the dependence of the pesticide penetration rate on the size of the used gold nanoparticles was established, which can be used to assess the toxicity of pesticides.

Agrochemical treatment of fields and sowing of contaminated seeds can also lead to pesticide contamination of agricultural soils. In addition, the leaching of pesticides from the soil by underground runoff and wind drift during plant pollination result in penetration of pesticides into natural waters. In this regard, it is also important to monitor pesticides permissible levels in aquatic and soil ecosystems. A number of studies have demonstrated the usage of SERS bio-substrates for the analysis of pesticides in agricultural soils and water bodies [98,99,100]. Thus, SERS bio-substrate based on gold nanoflowers obtained from biowaste was proposed for the analysis of glyphosate and its derivative aminomethylphosphonic acid in agricultural soils [99]. Biogeneration of the substrate based on corrugated plate-like porous nanostructures contributed to the high intensity of the SERS signal, which was successfully used for the direct detection of pesticides in the soil. Yao et al. developed a SERS bio-substrate based on superhydrophobic lotus leaf coated with silver nanoparticles for paraquat sensing with a detection limit of 1.2 μg/L [98]. Preliminary incubation of silver nanoparticles with target analyte and subsequent evaporation on the lotus leaf surface provides additional concentrating of the pesticide and its introduction into hot spots.

**Figure 3:**
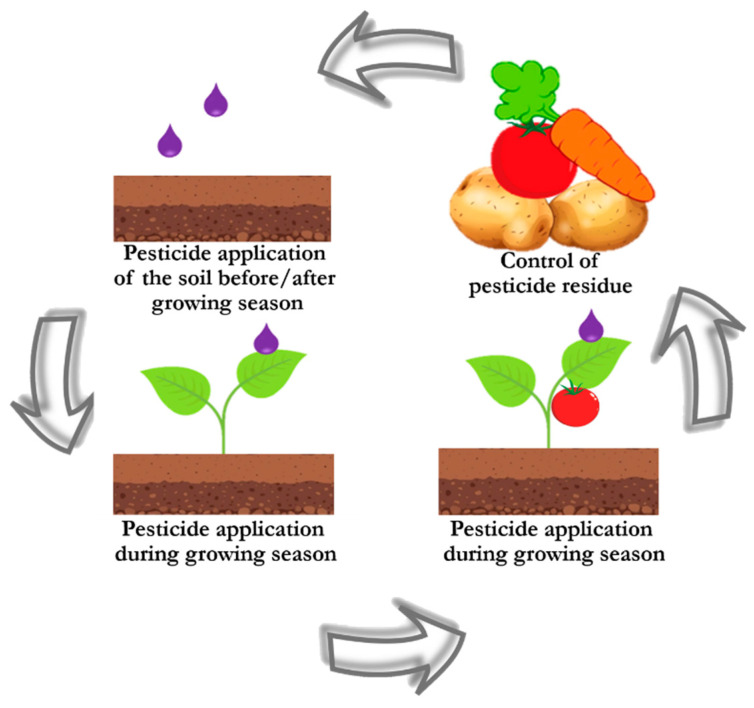
Stages of pesticides application and circulation.

Pesticides have become a hazard to the environment. Uncontrolled use of pesticides, as well as their use in large quantities, leads to contamination of soil and water, which ultimately harms its microflora/microfauna and prevents the absorption of important minerals by plants (Figure 3). Therefore, important tasks include monitoring the pesticides level in soil and water, as well as timely detection of pesticide residues on the surface of food products, the solution to which is the selection of suitable substrates that meet the requirements for the analysis of particular samples. Natural biomaterials such as leaves, wings, fish scales have unique micro/nanostructures and are suitable for the design and development of SERS substrates, as it was demonstrated by their successful applications for monitoring pesticides in agricultural products [85,94,95,101]. The biodegradability of composite SERS substrates based on natural biomaterials and biopolymers corresponds to current trends in green chemistry and is an additional advantage for future application of SERS substrates as sensors attached to food packaging (e.g., as SERS chips), ensuring safety in contact with food [29,102]. In addition, among the various application formats of natural SERS substrates, the flexible substrates have shown great promise due to their porous structure, which ensures the accumulation of target analyte without complicated sample preparation.

**Table 2:** Pesticides detection in various samples using bio-derived material-based SERS substrates.

Substrate	Plasmonic Nanostructures	Analyte	Detection Limit	Samples	Ref.
Natural biomaterials					
lotus leaf	AgNPs	paraquat	4.7 × 10^−9^ M	lake, tap and drinking waters	[98]
cicada wing	AgNPs	difenoconazole	3.9 × 10^−8^ M	potato	[103]
dragonfly wing	Au nanoislands	cypermethrin	10^−3^ ng/cm^2^	tomato peels	[85]
fish scale bio-wastes	Au nanoflowers	glyphosate	2.6 × 10^−6^ M	agricultural soil	[99]
		aminomethylphosphonic acid	2.4 × 10^−6^ M		
cicada wing	Au nanofilm	acephate	10^−9^ mg/mL	pear peels	[86]
lotus leaf mastoid	Ag micro/nanoarrays on PDMS film	thiram	10^−6^ M	dendrobium leaves and stem	[93]
		fonofos	10^−5^ M		
		triadophos	10^−7^ M		
capsicum, celery, cole	Ag nanoislands	paraquat	10^−9^ M	capsicum, celery, cole	[94]
		fenthion	10^−8^ M		
tea leaves	AuNPs	ferbam	-	tea leaves	[95]
soybean leaves	AuNPs	acetamiprid	4.5 × 10^−7^ M	soybean leaves	[96]
		chlorothalonil	3.7 × 10^−6^ M		
spinach leaf	AuNPs	dimethoate	4 × 10^−6^ M	spinach leaf	[97]
Biopolymer					
bacterial cellulose nanocrystal	AuNPs	thiram	1.5 × 10^−7^ M	peach juice	[90]
			2 × 10^−7^ M	apple juice	
			2 × 10^−7^ M	grape juice	
bacterial nanocellulose paper	AgNPs	methomyl	3.6 × 10^−7^ M	orange and apple peels	[81]
bacterial nanocellulose	Ag nanorods	thiram	10^−9^ M	grape	[87]
chitosan	AgNPs	thiram	3.2 × 10^−5^ M	water samples	[100]
cellulose	AgNPs	chlorfenapyr	2.5 × 10^−6^ M	-	[101]
bacterial nanocellulose	succulent-like Ag nanoflowers	thiram	10^−10^ M	apple	[88]
nanocellulose fiber	AgNPs	carbendazim	10^−8^ M	-	[104]
nanocellulose paper	Au-Ag bimetallic NPs	thiram	10^−6^ M	apple	[89]
chitosan foam	AgNPs	triazophos	3.2 × 10^−5^ M	-	[51]
alginate–chitosan porous gel	Ag nanocubes	thiram	1.43 × 10^−8^ M	apple	[75]
alginate hydrogel	Au@Ag NPs	thiram	10^−10^ M	fruit juices, apple peels and cabbage leaves	[64]
gelatine hydrogel	AgNPs	sodium diethyldithiocarbamate	10^−5^ M	-	[91]
jellylike nitrocellulose texture	AgNPs	thiram	0.5 ng/cm^2^	apple peels	[105]
gelatin gel	AgNPs	malachite green	10^−9^ M	lake water	[67]

## 4. Functionalization of SERS Substrates for Improving Detection Capabilities

A major drawback of SERS is the difficulty in detecting trace amounts of target compound in complex samples (e.g., food) since in this case the Raman signal of a particular analyte is masked by the signals of compounds present in the matrix [106]. In this regard, the selectivity of the SERS technique can be enhanced by functionalizing plasmonic nanostructures with receptor molecules such as aptamers. Aptamers are single-stranded DNA or RNA molecules that have a specific spatial structure and are therefore able to recognize particular molecules [107,108,109]. To obtain aptamers with desired properties, SELEX (systematic evolution of ligands by exponential enrichment) technology is used. Aptamers can be considered as analogs of monoclonal antibodies, which can have high affinity and specificity. At the same time, they have a number of important advantages over antibodies [109]. Their production is much simpler, cheaper and faster than the production of monoclonal antibodies. Unlike antibodies, which are characterized by a large size that does not allow controlling the distance between the target analyte and the SERS active surface, aptamers show exceptional advantages in smaller molecular size, closer distance to the SERS active surface, and sequence editing capabilities [110]. Therefore, aptamers are the most suitable receptor molecule for constructing a functionalized SERS substrate for direct extraction of target molecules from a complex organic media. In addition, the functionalized SERS substrates allow for the detection of pesticides with low affinity for the SERS substrate, such as organophosphorus and organochlorine pesticides [23].

Table 3 summarizes the functionalized SERS substrates currently applied for aptamer-based pesticide detection. Analysis of pesticides using aptamers is realized in two approaches: pre-incubation of the aptamer with the target analyte and interaction of the formed complex with a plasmonic substrate followed by SERS measurements [111,112,113,114,115], or measurement of the SERS signal after incubation of the target analyte with the aptamer-functionalized SERS substrate [116,117,118]. For example, Zhi et al. proposed a biosensing strategy based on the catalytic activity of gold nanocluster-doped MXeneTi_3_C_2_ nanosheets [113]. The assay principle combines a dual-mode catalytic reaction of mandelic acid-HAuCl_4_ reduction to form Au nanoparticles and the use of an aptamer-functionalized SERS substrate. The proposed SERS biosensor is characterized by a low detection limit of isocarbophos, estimated at 4.5 × 10^−14^ M. Li et al. coupled catalytic reduction of polyethylene glycol 400-Ag^+^ using Fe metal–organic framework-loaded liquid crystal 4-octoxybenzoic acid with aptamer-analyte interaction [114]. The proposed amplified SERS assay allowed achieving a sensitivity of 0.010 nM for isocarbophos detection and was successfully applied for testing rice samples. Kamkrua et al. combined advances in nano-engineering of SERS substrates with an aptamer specific for paraquat to produce a functionalized SERS chip [116]. The authors showed that the use of the aptamer improved the sensitivity and selectivity of pesticide detection in natural water samples with a calculated detection limit of 0.1 μM. In addition, Barahona et al. demonstrated extraction capability of polymer-Au nanoparticle-aptamer composite microspheres for SERS detection of malathion [119]. The designed SERS substrate allowed label-free detection of malathion with a detection limit of 3.3 µg/mL.

## 5. The Potential of Raman Spectroscopy in Detecting Photosynthesis Inhibitor Substances Using Biomaterials

**Figure 4:**
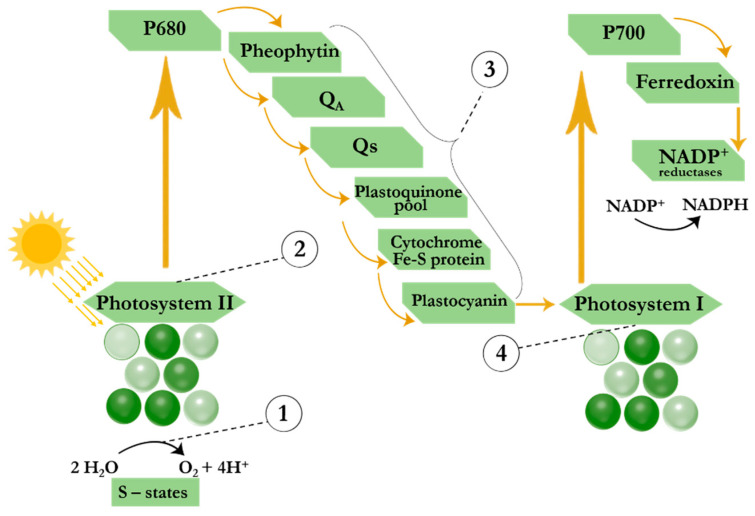
Scheme of the photosynthetic process and modes of action of herbicides on photosynthesis. (1) phenolic herbicides; (2 and 3) herbicides from groups of triazine, uracil, carbamate, pyridazinone, urea, amide, nitrile, phenylpyridazine and benzothiadiazinone; and (4) bipyridylium herbicides.

Some of the first commercialized pesticides were synthetic herbicides that inhibited the process of photosynthesis, thereby reducing the availability of nutrients for plant growth [119]. Such herbicides include phenylurea derivatives (diuron, linuron, etc.), triazine herbicides (atrazine, simazine, prometryn, propazine, etc.), herbicides from the uracil group (bromacyl, lenacil, etc.) and substituted phenols (ioxinil, bromoxynil, etc.) and others. Through photosynthesis (Figure 4), plants convert carbon dioxide and water into glucose using light energy, producing oxygen as a byproduct [120]. Photosynthesis occurs in the chloroplasts of plants, which contain energy-harvesting pigments such as chlorophyll A and B, xanthophylls, and carotenoids [121]. Light-harvesting complexes absorb excitation light, followed by the migration of excitation energy to the reaction centers of the photosynthetic apparatus. The energy of electronic excitation of the pigments of the light-harvesting complexes is used to activate the reaction centers of the photosystems and maintain electron transfer across the thylakoid membrane of chloroplasts. In the reaction center of photosystem II (PS II or P_680_), excitation of the chlorophyll molecule and electron transfer from the water molecule to the acceptor plastoquinone Q_a_, bound on the stromal side of the membrane in the subunit of the integral protein D_2_, occur. The electron is then transferred to the secondary plastoquinone molecule Q_b_, which forms fully reduced plastoquinol molecules (Q_b_H_2_). The plastoquinol molecules release their binding site and diffuse within the lipid bilayer of the thylakoid membrane, providing electron transfer from the cytochrome complex to the oxidized centers of the second photosystem (PS I or P_700_). Thus, energy-rich compounds such as ATP and NADP^+^ are synthesized [121]. Impacts of pesticides on the photosynthetic apparatus are summarized in the reviews [122,123] and are schematically shown in Figure 4. Thus, PSII-inhibiting herbicides block electrons transfer from PS II to PS I via binding to the Q_B_-binding site of D_1_ protein. Blockage of electron transport in D1 leads to various cellular dysfunctions such as inhibition of CO_2_ fixation and D1 turnover, accumulation of NO content in chloroplasts, loss of carotenoids and ascorbate, and decreased chlorophyll content [124]. The excitation energy that does not participate in the charge separation is dissipated through various relaxation processes (fluorescence, heat, etc.), among which an easily measurable effect is a significant increase in chlorophyll fluorescence.

The recording of changes occurring in photosynthetic systems during the interaction of toxic pesticides with photosynthetic enzymes has formed the basis for the development of various biosensors with optical (measuring changes in chlorophyll fluorescence) or electrochemical signal detection [125,126,127,128,129,130]. Here, whole cells or isolated cellular components are used as receptor molecules to achieve specific detection of pesticides. For example, Varsamis et al. used photosynthetic membranes isolated from higher plants and photosynthetic microorganisms as biorecognition elements in a microfluidic sensor for the detection of herbicides by chemiluminescent analysis of hydrogen peroxide produced by thylakoid membranes [131]. In addition, a self-powered biosolar herbicide sensor was developed based on thylakoid membranes immobilized on the anode for direct photoelectrocatalysis [132]. The principle of the biosensor is based on the inhibition of thylakoid photosystems by herbicides, leading to a decrease in the output current. Measuring the output current allows determining the concentration of the target analyte. Note that isolated supramolecular complexes of PS II allow for greater sensitivity compared to whole cells, since they lack protective mechanisms against herbicides [130]. However, despite attempts to improve the throughput [133] by preconcentration of samples, stabilization of photosynthetic complexes, advances in nanofabrication techniques, as well as assay design, the achieved performance of biosensors based on photosynthetic enzymes often does not meet the criteria for maximum residue levels of pesticides in food samples established by European regulatory. Therefore, improving systems where photosynthetic organisms are used as receptor molecules remains a challenge for future research.

Until now, most studies have focused mainly on the development of SERS substrates by testing pure pesticide solutions. SERS detection of pesticides in food matrices requires a relatively complex extraction procedure. However, the availability of simple and effective methods for in-field identification of pesticides could be of great interest for public health and for proper monitoring of agricultural products, as well as for the assessment of water and soil pollution. In this regard, the unique sensitivity of the SERS methods, which allows differentiation of areas with different chemical compositions and structures to study processes in plant tissues and cells, as well as on the surface of plants, can be considered as a potential tool for in situ and non-destructive assessment of contamination. For example, SERS imaging of plant tissues allows real-time monitoring and detection of contamination localization on their surface or inside [134,135]. Thus, Yang et al. treated tomato with thiabendazole and after a certain period carried out direct SERS measurements of tomato leaf and flower tissues after the application of gold nanoparticles [136]. The results of SERS imaging determined the times during which the pesticide accumulated in tomato leaves. In addition, SERS mapping was effective in determining the distribution of thiabendazole in tomato leaf trichomes. After 4 days, an additional SERS signal was detected at 737 cm^−1^, presumably related to the adenine content of the sample, which may indicate the plant response to stress as a result of pesticide exposure. These results demonstrate the potential of the SERS methods to detect pesticides directly in biomaterials where the toxicant accumulates. Above, there are several studies where plant leaves were used both as a substrate after direct application of SERS-active silver nanoparticles and as a bioreceptor system due to the accumulation of pesticides [93,94,95,96,97]. However, the rapid degradation of target analytes to derivatives, stimulated by silver nanoparticles under SERS measurement conditions, prevents this approach from being considered as a quantitative analysis of pesticides [137]. In addition, it is worth noting that pure natural material itself can have its own Raman signal and luminescence [138], which, under the conditions of SERS measurements, can interfere with the SERS spectrum of the target analyte.

Another potential approach to identifying plants with excessive herbicide levels is to combine fluorescence imaging with Raman spectroscopy. Fluorescence imaging allows for the assessment of photosynthesis efficiency, while Raman spectroscopy provides information on the presence and content of various specific compounds on the surface and inside plants [139]. Thus, Vítek et al. studied changes in pigment composition, photochemistry of PSII and non-photochemical quenching using fluorescence imaging and Raman spectroscopy after treatment of sunflower leaves with herbicides inhibiting photosystem enzymes [140]. It was shown that after herbicide application, the content of phenolic compounds increased, while the Raman intensity ratio of carotenoid/chlorophyll decreased. In addition, the quantum efficiency of PSII and non-photochemical quenching, estimated by chlorophyll fluorescence, significantly decreased when plants were treated with herbicides from the group of carotenoid biosynthesis inhibitors. In a later study Vitek et al. shown that after application of the herbicide metribuzin (an inhibitor of PS II), high-resolution resonance Raman images showed areas of local increase in the carotenoid signal, indicating activation of defense mechanisms [141]. Moreover, the recorded shift of the carotenoid band may be associated with structural changes in the carotenoids. In summary, this study demonstrates the potential of Raman spectroscopy in detecting herbicides that inhibit plant biosynthesis by detecting changes in pigment composition and secondary biosynthetic metabolites (phenolics), and combining this method with fluorescence imaging allows for a complete assessment of biochemical and functional changes in leaves after herbicide exposure [140,141].

Singh et al. used Raman spectroscopy to distinguish three different glyphosate-resistant and four glyphosate-susceptible populations of Palmer amaranth to assess herbicide tolerance and stress response in plants under field conditions [142]. It was found that changes in the intensity of vibrational bands at 1156, 1186, and 1525 cm^−1^, corresponding to carotenoids, differentiated between herbicide-treated and untreated susceptible populations. The study showed that Raman spectroscopy allows identification of treated and untreated susceptible populations with high accuracy (90 and 73.3%, respectively).

In summary, the success in applying Raman spectroscopy to studies of photosynthetic pigment–protein complex and the reduction of pigment fluorescence by using SERS-active metal nanoparticles [143] makes this approach promising for further development of sensors for in situ detection of plant photosynthesis-inhibitory compounds, including herbicides.

## 6. Conclusions and Outlook

The ease of measurement and analysis, which provides rich structural information without preliminary labeling processes and complex sample preparation, has led to increased interest and significant expansion of surface-enhanced Raman spectroscopy for pesticide detection. In addition, the important advantage of the SERS method, which has a high response rate, provides the convenience of using this technique on site through the development of portable devices. In this review, we summarized the recent studies (mainly over the past 10 years) aimed at developing SERS substrates based on various bio-derived materials, where:

- The unique structure, natural pattern, surface hydrophobicity and gaps provided by natural materials form many “hot spots” and create the necessary conditions for the detection of trace pesticides;
- Biopolymer-based SERS substrates, combining flexibility, stability and low costs, demonstrate high sensitivity and allow pre-concentration of pesticides directly from the sample;
- Functionalization of SERS substrates extracts target analytes from the complex organic sample environment and enhance the selectivity of pesticide analysis.

Although novel biocompatible SERS substrates based on natural biomaterials and biopolymers have demonstrated desirable analytical performance in the detection of pesticides in laboratory studies, the translation from research to practical applications remains a challenging task, since the basic requirements for this include scale-up fabrication, unification of properties and long-term stability. The following additional specific requirements for the SERS sensors should also be considered:

- Despite the unique surface morphology of natural biomaterials (petals, eggshells, leaves, cicada wings, etc.), which provide hierarchical micro/nanostructures with a large number of gaps in the designed substrates to enhance the electromagnetic field, as well as the natural hydrophobicity of materials, mechanical strength, durability and repeatability of SERS substrates are the weak points of natural biomaterials. Inspired by the pattern and morphology of the surface of natural biomaterials and considering their weaknesses when designing SERS substrates, scientists obtain multifunctional flexible SERS substrates with replicated micro/nanostructure of natural materials, for example, using nanoimprinting technology [144,145].
- A common drawback of all types of SERS substrates in the direct analysis is the presence of compounds that contribute to the recorded spectrum and complicate the interpretation of the assay results. The solution to this problem is the functionalization of SERS substrates with aptamers, antibodies or other receptors to enhance the substrate specificity [111].
- Fluorescence arising from close interactions between plasmonic nanoparticles and the target molecule or causing by the analysis of fluorophore-containing samples is a competing effect that suppresses the Raman signal and reduces the sensitivity of sensing [146,147]. To improve the performance of SERS substrates, it is necessary to design substrates based on bio-inspired materials using components that inhibit fluorescence while maintaining or even increasing achievable EF values. For example, a graphene-containing composite SERS substrate provides improved SERS efficiency through a chemical enhancement mechanism in addition to reducing the fluorescence background [147].
- The disadvantage of flexible SERS substrates is the low accuracy of testing, since some pesticides penetrate deeply into the sample tissue and soft treatment of the surface is insufficient. In this case, deep extraction procedures are still required for sample preparation. In this regard, the development of a hybrid substrate combining a flexible SERS substrate with separation methods including microfluidic systems and thin layer chromatography allows for the sensing procedure to be simplified and its efficiency to be increased [148,149,150].
- The uniformity and reproducibility of SERS substrates are key parameters determining the practical applicability of the method, but achieving these characteristics remains a challenge. Standard methods such as incorporation of plasmonic nanoparticles into the structure of flexible substrates or in situ growth of nanoparticles on the surface of bio-derived materials do not provide homogeneity. Therefore, new fabrication techniques are still in demand.
- The standard analysis procedure consists of depositing a probe molecule on a bio-derived-based SERS substrate and disposing of the substrate after measuring the Raman spectra. Fabrication of reusable SERS substrates in a low-cost and easy-to-use manner is a relatively new direction that presents favorable opportunities for sensing applications. The proposed approaches to ensure reusability of SERS substrates include rinsing the analyte from the surface using solvents and degradation or decomposition of the probe molecule using various techniques (e.g., ultrasound, plasma cleaning, catalytic degradation) [151].
- Further application of SERS substrates for monitoring agricultural products requires engineering of portable and handheld Raman systems. In the field, it is important to quickly assess pesticide levels outside the laboratory to detect when toxicants reach dangerous concentrations in a timely manner. The use of portable SERS spectrometers at the sampling site requires large-scale production of reproducible SERS substrates [152,153]. In addition, the design of portable spectrometers should be improved to variability of the excitation laser wavelength range (from visible to near infrared). Finally, the use of SERS substrates in combination with a portable recording device for on-site pesticide analysis requires the expansion of the database/library with spectra of all pesticides.

**Figure 5:**
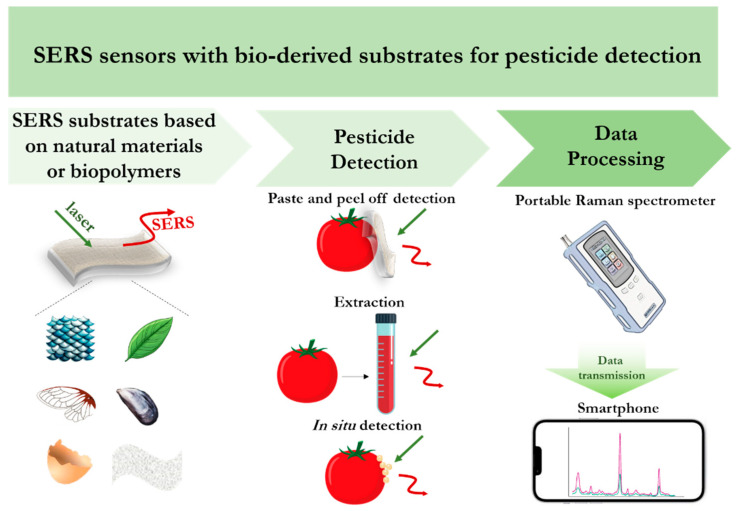
Current strategies of SERS sensing of pesticides using bio-derived substrates.

Figure 5 shows the current SERS sensing strategies for the timely detection of pesticides in water or soil samples, and agriculture products, the practical implementation of which is facilitated by the improved performance of the above-mentioned SERS sensors and the advances in sensor design. And the combination of flexible substrates with compact Raman spectrometers opens up the possibility of using portable sensors as effective analytical tools for on-site pesticide detection.

In addition, action of pesticides on the photosynthetic apparatus of plants underlies numerous developments of biosensors with fluorescence and electrochemical detection. Current in situ SERS sensors are limited by the format when a layer of plasmonic nanostructures is applied to plant leaves after a certain period of time after exposure to a pesticide, and direct SERS measurements of herbicide are carried out. However, several studies showed changes in the composition of pigment systems after herbicide treatment using Raman spectroscopy. These studies demonstrate the high potential of isolated supramolecular photosynthetic complexes as bioreceptors that can be used in SERS sensors for pesticide detection.

## Figures and Tables

**Table 1:** The diversity of SERS substrates based on bio-derived materials.

Substrate	Plasmonic Nanostructures	Raman Reporter Molecule	Limit of Detection, M	RSD of SERS Intensity, %	Ref.	
Natural biomaterials						
*Canna generalis* leaf	Au film and AuNPs	Rh6G	10^−5^	-	[37]	
cicada wings	CuNPs	crystal violet (CV)	10^−7^	16	[38]	
fish scale substrate	AgNPs	perfluorooctane sulfonamide	10^−7^	6.4	[39]	
mussel shell	Au@Ag NPs	Rh6G	10^−9^	6.5	[40]	
cicada wings	Ag-coated Au nanocubes	Rh6G	5 × 10^−9^	8.2	[41]	
*Mytilus coruscus*	graphene oxide-Ag NPs	Rh6G	10^−9^	6.6	[42]	
chicken eggshell	AuNPs	Rh6G	10^−8^	10.056–11.924	[43]	
diatom frustule	AuNPs	malachite green	10^−9^	-	[44]	
Biopolymers						
cellulose aerogel	ZnO@Ag NPs	Rh6G	10^−10^	-	[45]	
silk nanoribbons	AuNPs	4-Aminothiophenol (4-ATP)	10^−15^	11.2	[46]	
filter paper coated with chitosan and alginate	AuNPs	4-mercaptobenzoic acid	1.37 × 10^−12^	8.2	[47]	
chitosan	Ag spheres	p-ATP	10^−4^	-	[48]	
	Ag nanocubes	p-ATP	10^−9^	26.11		
	Au nanospheres	p-ATP	10^−5^	-		
	Au nanorods	p-ATP	10^−4^	-		
chitosan	AgNPs	methylene blue	1.6 × 10^−9^	5.2	[49]	
chitosan	AuNPs	4-MBA	10^−8^	5.66	[50]	
chitosan foam	AgNPs	Nile blue A	3 × 10^−11^	16.4	[51]	
		Rh6G	2 × 10^−7^	-		
methylcellulose	AgNPs	Nile blue A	10^−8^	7.47–9.95	[52]	
cellulose nanofibers	AgNPs	4-ATP	8 × 10^−5^	-	[53]	
cellulose nanofibrils-coated filter paper	AgNPs	4-ATP	1 × 10^−10^	9	[54]	
cellulose nanofibers deposited on quartz paper	AgNPs and Au nanostars	4-ATP	8 × 10^−8^	-	[55]	
cellulose paper	AgNPs	4-ATP	41 × 10^−9^	17.7	[56]	
cellulose aerogel	ZnO@Ag NPs	Rh6G	10^−10^	-	[45]	
bacterial cellulose hydrogel	AuNPs	Rh6G	10^−10^	-	[57]	
cellulose acetate hydrogel	cauliflower-like AuNPs	MB	10^−12^	-	[58]	
fungal β-D-glucan, botryosphaeran	AgNPs	CV	1.2 × 10^−11^	-	[59]	
cotton swabs	Ag nanoflowers	carmine	10^−8^	11.2	[60]	
calcium alginate sponge	Au nanorods	Rh6G	0.1× 10^−9^	7.94	[61]	
calcium alginate gel beads	Au nanobipyramids	Rh6G	0.4× 10^−9^	6.57	[62]	
calcium alginate fiber	AuNPs	CV, Rh6G	10^−8^, 10^−9^	-	[63]	
sodium alginate hydrogels	Au@Ag NPs	4-mercapto- benzoic acid	1 × 10^−10^	3.56	[64]	
hyaluronic acid microgel	Au nanobipyramids@Ag	Rh6G	1 × 10^−9^	2.82	[65]	
alginate/gelatin hydrogel	AuNPs	4-mercaptophenyl-boronic acid	10^−8^	-	[66]	
gelatin gel	AgNPs	Rh6G	10^−9^	3.45	[67]	

**Table 3:** Functionalized SERS substrates for aptamer-based pesticide detection.

Functionalized SERS Substrate	Analyte	Limit of Detection	Samples	Ref.
AuNPs modified by aptamer PQ77-SH	paraquat	0.10 × 10^−6^ M	natural water	[116]
PCR sealing membranes dotted with AgNPs and aptamer	acetamiprid	10^−8^ M	N/A	[111]
Ag dendrites modified by thiolated aptamer	isocarbophos	3.4 × 10^−6^ M	apple juice	[117]
	omethoate	2.4 × 10^−5^ M		
	phorate	4 × 10^−7^ M		
	profenofos	1.4 × 10^−5^ M		
Au-doped fullerene carbon dots combined with chlorpyrifos aptamer	chlorpyrifos	2.40 × 10^−7^ mg/kg	tea	[112]
Au nanocluster doped nanosheets sol coupled with aptamer	isocarbophos	4.5 × 10^−14^ M	water	[113]
Fe metal–organic framework-loaded liquid crystal 4-octoxybenzoic acid coupled with bimodal nanosilver modified by aptamer	isocarbophos	10^−11^M	rice	[114]
AgNPs modified by aptamer	malathion	5 × 10^−7^ M	tap water	[115]
AuNPs-doted polymer particles modified by thiolated aptamer	malathion	10^−5^M	N/A	[118]

## Data Availability

No new data were created or analyzed in this study. Data sharing is not applicable to this article.
